# A chromosomal microarray analysis-based laboratory algorithm for the detection of genetic etiology of early pregnancy loss

**DOI:** 10.3389/fgene.2023.1203891

**Published:** 2023-06-29

**Authors:** Na Liao, Zhu Zhang, Xijing Liu, Jiamin Wang, Rui Hu, Like Xiao, Yunyuan Yang, Yi Lai, Hongmei Zhu, Lingping Li, Shanling Liu, He Wang, Ting Hu

**Affiliations:** ^1^Department of Medical Genetics, West China Second University Hospital, Sichuan University, Chengdu, Sichuan, China; ^2^Department of Obstetrics and Gynecology, West China Second University Hospital, Sichuan University, Chengdu, Sichuan, China; ^3^Key Laboratory of Birth Defects and Related Diseases of Women and Children (Sichuan University), Ministry of Education, Chengdu, China

**Keywords:** chromosomal abnormality, chromosomal microarray analysis, copy-number variation, uniparental disomy, miscarriage, pregnancy loss, products of conception, first trimester

## Abstract

**Background:** Chromosomal abnormalities are a major cause of early pregnancy loss. However, models synthesizing existing genetic technologies to improve pregnancy outcomes are lacking. We aim to provide an integrated laboratory algorithm for the genetic etiology of couples who experienced pregnancy loss.

**Methods:** Over a 6-year period, 3,634 products of conception (POCs) following early pregnancy loss were collected. The clinical outcomes from a laboratory algorithm based on single nucleotide polymorphism (SNP) array, fluorescence *in situ* hybridization (FISH), and parental chromosomal karyotyping assays were comprehensively evaluated.

**Results:** In total, 3,445 of 3,634 (94.8%) POCs had no maternal-cell contamination. Of those POCs, the detection rate of abnormal results was 65.2% (2,247/3,445), of which 91.2% (2,050/2,247) had numerical chromosomal abnormalities, 2.7% (60/2,247) had copy-number variations (CNVs) ≥10 Mb, 2.7% (61/2,247) had CNVs of terminal deletion and duplication, 2.8% (62/2,247) had CNVs <10 Mb, and 0.6% (14/2,247) had uniparental disomy. Furthermore, FISH confirmed 7 of the 60 POCs with mosaic aneuploids below 30% based on the SNP array results as tetraploid. Of the 52 POCs with CNVs of terminal deletion and duplication, 29 couples had balanced rearrangements based on chromosomal karyotyping.

**Conclusion:** The integrated SNP array-based algorithm combined with optional FISH and parental chromosomal karyotyping is an effective laboratory testing strategy, providing a comprehensive and reliable genetic investigation for the etiology of miscarriage, regardless of the number of miscarriages and the method of conception.

## Introduction

Pregnancy loss is the most common pregnancy-related complication and the risk of miscarriage is approximately 10%–15% in all clinically recognized pregnancies, most of which occur during the first trimester (Rai and Regan, 2006; Dhillon et al., 2014; Colley et al., 2019). Of these early pregnancy losses, 50%–70% are caused by chromosomal abnormalities, including numerical and structural abnormalities (Hassold et al., 1980; Menasha et al., 2005). Specifically, balanced structural chromosomal abnormalities, mostly balanced reciprocal or Robertsonian translocations, are observed in approximately 2%–5% of couples who experienced pregnancy loss (Practice Committee of the American Society for Reproductive Medicine, 2012). Antiphospholipid syndrome, prothrombotic states, uterine anomalies, maternal endocrine disorders, psychological factors, and lifestyle are also associated with pregnancy loss (Practice Committee of the American Society for Reproductive Medicine, 2012).

Genome-wide, high-resolution molecular technologies, including chromosomal microarray analysis (CMA) and next-generation sequencing (NGS), have become broadly implemented. Consequently, pathogenic/likely pathogenic (P/LP) copy-number variations (CNVs) have been identified in approximately 15%–30% of patients with neurodevelopmental disorders and 6% of fetuses with structural anomalies observed by ultrasound (Manning et al., 2010; Miller et al., 2010; Wapner et al., 2012). Thus, a CNV assessment is the first-tier recommendation for these conditions (Miller et al., 2010; American College of Obstetricians and Gynecologists Committee on Genetics, 2013). Although several studies have explored the clinical utility of CMA or NGS in products of conception (POCs) (Zhang et al., 2009; Li et al., 2013; Kooper et al., 2014; Shen et al., 2016), until 2016, the Society for Maternal-Fetal Medicine recommended against using CMA as a first-line test to evaluate first-trimester pregnancy loss due to limited data (Society for Maternal-Fetal Medicine Dugoff et al., 2016).

Several large cohort studies have recently evaluated the diagnostic yield of different chromosomal testing techniques (Shearer et al., 2011; Levy et al., 2014; Rosenfeld et al., 2015; Fan et al., 2020; Wang et al., 2020). However, models synthesizing existing genetic technologies to improve pregnancy outcomes are lacking. Therefore, we performed a large-scale retrospective study to build an integrated CMA-based laboratory algorithm for the genetic etiology of early pregnancy loss, regardless of the number of miscarriages and the method of conception. We also aimed to provide practical genetic counseling recommendations for couples who have experienced pregnancy loss.

## Materials and methods

### Specimen collection

From January 2016 to October 2021, POCs of early pregnancy loss prior to 13 gestational weeks were collected for this 6-year retrospective study at a tertiary-level referral center (West China Second University Hospital, Sichuan University). Trained clinical geneticists performed pretest counseling for parents who intended to seek a possible genetic etiology. Before testing, written informed consent was obtained from all couples agreeing to undergo a single nucleotide polymorphism (SNP) array analysis, and consecutive parental consent was obtained in instances of positive POC results. Those with a pregnancy history were recruited regardless of the conception method (natural conception or assisted reproductive technology [ART]). The Medical Ethics Committee of West China Second University Hospital approved the study design.

POCs obtained by suction curettage or spontaneous passage were collected in 15 mL conical tubes with saline solution and stored at 4°C. A saline solution rinse was performed three or more times; then, chorionic villi were dissected from the maternal decidua using forceps and scissors and divided into two samples, if necessary: one for CMA and one for consecutive fluorescence *in situ* hybridization analysis (FISH).

### DNA isolation

Whole-genome DNA was extracted from POC samples using a QIAamp DNA Mini Kit (Qiagen, Valencia, CA, United States). A NanoDrop One microvolume UV-Vis spectrophotometer (Thermo Fisher Scientific, Madison, GA, United States) was used to determine the DNA’s quantity and quality. DNA denaturation was confirmed via electrophoresis on a 1% agarose gel. Poor-quality DNA samples were excluded.

### CMA

Whole-genome DNA was subjected to SNP array analysis using the CytoScan 750 K Array (Thermo Fisher Scientific, Santa Clara, CA, United States). The GRCh38 (hg18) genome was used for annotation. CNVs >100 kb or those that affected >50 contiguous probes were considered. Terminal regions of homozygosity (ROHs) > 5 Mb or interstitial ROHs >10 Mb were analyzed using the uniparental disomy (UPD) tool_0.2 software to separate the results into UPD or consanguinity based on comparisons with the parental results as described in our previous study (Hu et al., 2021).

The chromosomal abnormalities detected by the SNP array were classified into five groups: 1) numerical chromosomal abnormalities, including single aneuploidy, multiple aneuploidy, mosaic aneuploidy, and polyploidy; 2) CNVs ≥10 Mb (large CNVs), those with gains or losses of chromosome regions >10 Mb; 3) CNVs of terminal deletion and duplication, those with one deletion and one duplication at the ends of different chromosomes or the same chromosome; 4) CNVs <10 Mb (submicroscopic CNVs), those with gains or losses of chromosome regions <10 Mb; and 5) UPD, those with both homologous chromosomes inherited from one parent based on a comparison with the parental SNP array results.

Significant maternal cell contamination (MCC) was defined as levels of maternal cells exceeding 30%, tested directly by an SNP array (Zahir and Marra, 2015); patients with significant MCC were excluded.

### Quantitative fluorescent polymerase chain reaction (QF-PCR)

The SNP array limit of detection for low-level mosaicism is 10%–20% (Cross et al., 2007). Therefore, if the MCC below 30% was suspected by the SNP array, QF-PCR of the chorionic villi and maternal peripheral blood was subsequently performed to validate the proportion of maternal DNA in female embryos.

QF-PCR was performed using 20 highly polymorphic short tandem repeat markers, including four for chromosome 21 (D13S628, D13S742, D13S634, and D13S305), four for chromosome 18 (D18S1002, D18S391, D18S535, and D18S386), six for chromosome 21 (D21S1433, 21q11.2, D21S1411, D21S1414, D21S1412, and D21S1445), and six for sex chromosomes (AMXY, DXS1187, DXS8377, DXS6809, DXS981, and SRY).

### FISH analysis

Mosaicism for CNVs ≥5 Mb was tested directly by SNP array when the proportion exceeded the 30% detection threshold. If the SNP array mosaicism result was below 30%, FISH was simultaneously performed for confirmation, which could also detect potential tetraploids as incidental findings.

AneuVysion probe sets (Vysis/Abbott, Downers Grove, IL, United States) that probed for chromosomes 2, 4, 5, 7, 9, 12, 13, 15, 16, 17, 18, 21, 22, X, and Y were used for hybridization to the chorionic interphase cells of POCs according to the manufacturer’s instructions.

### Chromosomal karyotyping

When CNVs of terminal deletion and duplication or trisomy associated with acrocentric chromosomes (13, 14, 15, 21, and 22) were detected in the POCs, peripheral blood samples from the couple were karyotyped by traditional cytogenetic G-band analysis with a resolution of 450–550 bands to confirm whether one parent had reciprocal translocations, inversions, or Robertsonian translocations.

### Statistical analyses

Statistical analyses were performed using SPSS software v24.0 (IBM Corp., Armonk, NY, United States). Continuous variables were compared using Student’s t-test, and categorical variables were compared using chi-square or Fisher’s exact analyses, as appropriate. Data are presented as means ± standard deviations. A logistic regression analysis was also conducted with adjustments for potential confounding effects. A *p*-value of <0.05 was considered statistically significant.

## Results

### Specimen characteristics and general findings

Initially, 3,634 POCs were recruited for this study; 189 (5.20%) samples were excluded for significant MCC (38 samples) or poor DNA quality (151 samples); thus, 3,445 samples were included in the analyses. In total, 57 samples had a suspected MCC below 30% by SNP array and underwent QF-PCR analysis. In addition, 46 samples were confirmed to have 10%–29% MCC, which did not influence the results; thus, they were not excluded. The testing turnaround time ranged from 3 to 7 days (4.36 ± 1.79 days). The maternal ages ranged from 19 to 46 years (31.04 ± 4.30 years), and the number of pregnancies lost ranged from 1 to 6 (1.73 ± 0.92 times), including 2,711 (78.7%) cases with recurrent miscarriage (RM). Additionally, 1,746 pregnancies developed via ART (50.7%).

Overall, 1,198 (34.8%) cases had normal results, 2,247 (65.2%) had abnormal results, of which 2,050 (91.2%) had numerical chromosomal abnormalities, 60 (2.7%) had CNVs ≥10 Mb (excluding four combined with numerical abnormalities), 61 (2.7%) had CNVs of terminal deletion and duplication (excluding five combined with numerical abnormalities), 62 (2.8%) had CNVs <10 Mb (excluding 28 combined with numerical abnormalities), and 14 (0.6%) had UPD (excluding four combined with numerical abnormalities) (Table 1).

**TABLE 1:** Summary and characterization of chromosomal abnormalities identified by CMA in 3445 POC samples.

	n (%)			Maternal age (mean ± SD)	Miscarriages (mean ± SD)	ART (n (%))	RM (n (%))
Single aneuploidy	1,530 (44.4)			31.60 ± 4.60	1.67 ± 0.90	824 (53.9)	1,231 (80.5)
trisomy		1,279 (83.6)					
chr 2			40 (3.1)				
chr 3			27 (2.1)				
chr 4			49 (3.8)				
chr 5			16 (1.3)				
chr 6			22 (1.7)				
chr 7			30 (2.3)				
chr 8			42 (3.3)				
chr 9			26 (2.0)				
chr 10			29 (2.3)				
chr 11			20 (1.6)				
chr 12			16 (1.3)				
chr 13			75 (5.9)				
chr 14			44 (3.4)				
chr 15			93 (7.3)				
chr 16			412 (32.2)				
chr 17			11 (0.9)				
chr 18			21 (1.6)				
chr 19			1 (0.1)				
chr 20			28 (2.2)				
chr 21			80 (6.3)				
chr 22			197 (15.4)				
tetrasomy		2 (0.1)					
chr 13			1 (50.0)				
chr 15			1 (50.0)				
monosomy		249 (16.3)					
chr 21			13 (5.2)				
chr X			236 (94.8)				
Multiple aneuploidy	111 (3.2)			31.96 ± 5.36	1.75 ± 0.96	59 (53.2)	89 (80.2)
Mosaic anueploidy	125 (3.6)			31.35 ± 3.89	1.45 ± 0.58	74 (59.2)	93 (74.4)
Polyploidy	284 (8.2)			30.35 ± 3.89		115 (40.5)	258 (90.8)
triploidy		277 (97.5)					
tetraploidy		7 (2.5)					
CNVs> 10 Mb	60 (1.7) [4]*			30.84 ± 3.41	1.75 ± 0.83	30 (47.6)	53 (84.1)
P CNVs		38 (63.3)					
LP CNVs		20 (33.3) [3]*					
VUS		2 (3.3) [1]*					
CNVs of terminal deletion and duplication	61 (1.8) [5]*			29.42 ± 3.37	1.72 ± 0.72	26 (44.1)	50 (84.7))
≥10 Mb		40 (65.6) [2]*					
<10 Mb		21 (34.4) [3]*					
CNVs<10 Mb	62 (1.8) [28]*			30.07 ± 4.40	1.97 ± 0.96	19 (30.6)	55 (88.7)
P CNVs		21 (33.9) [11]*					
LP CNVs		-					
VUS		41 (66.1) [17]*					
UPD	14 (0.4) [4]*			29.31 ± 3.00	1.60 ± 1.02	10 (71.4)	8 (57.2)
Normal	1,198 (34.8)			30.52 ± 3.86	1.81 ± 0.99	589 (49.2)	874 (73.0)
Total	3,445 (100.00)			31.08 ± 4.31	1.60 ± 0.92	1746 (50.7)	2,711 (78.7)

CMA: chromosomal microarray analysis; POC: products of conception; SD: standard deviation; ART: assisted reproductive technology; RM: recurrent miscarriage; P CNVs: pathogenic copy number variations; LP CNVs: likely pathogenic copy number variations; VUS: variations of uncertain significance; UPD: uniparental disomy.

[]*: cases combained with numerical abnormalities.

The maternal age was significantly higher for those with abnormal results than for those with normal results (31.32 ± 4.49 vs. 30.52 ± 3.86, *p* < 0.001), and participants with numerical chromosomal abnormalities were significantly older than those with other abnormalities (31.43 ± 4.53 years vs. 30.07 ± 3.68 years, *p* < 0.001). In contrast, participants with abnormal results had fewer lost pregnancies than those with normal results (1.69 ± 0.88 vs. 1.81 ± 0.99, *p* = 0.025). However, the number of lost pregnancies did not differ between participants with numerical abnormalities and those with other abnormalities (1.68 ± 0.87 vs. 1.79 ± 0.88, *p* = 0.241). In addition, the RM proportion did not differ between participants with abnormal and normal results (48.2% [1,084/2,247] vs. 50.8% [609/1,198], *p* = 0.147) or between participants with numerical and other abnormalities (52.4% [1,074/2,050] vs. 45.7% [90/197], *p* = 0.072). The proportion of those conceived via ART did not differ between participants with abnormal and normal results (51.5% [1,157/2,247] vs. 49.2% [589/1,198], *p* = 0.194), but significantly more for participants with numerical abnormalities than those with other abnormalities (52.3% [1,072/2,050] vs. 43.1% [85/197], *p* = 0.014). However, when maternal age was considered, the difference was statistically significant (*p* = 0.417).

### Numerical chromosomal abnormalities

Most of the numerical chromosomal abnormalities (2,050/2,247 [91.2%]) were single aneuploids (1,530/2,247 [68.1%]). Trisomy was the most common abnormality, occurring in 1,279 of 1,530 cases (83.6%), followed by monosomy (249/1,530 [16.3%]) and tetrasomy (2/1,530 [0.1%]). Aneuploidy was identified in every chromosome except chromosome 1; the most common was trisomy 16 (412/1,530 [26.9%]), followed by monosomy X (236/1,530 [15.4%]) and trisomy 22 (197/1,530 [12.9%]) (Table 1).

Of those with abnormal results, 4.9% (111/2,247) had multiple aneuploids, of which 92.8% (103/111) had double aneuploids. Furthermore, 5.6% (125/2,247) had mosaic aneuploids, of which 48.0% (60/125) were below 30%, including seven cases combined with the following: CNVs ≥10 Mb (n = 3), CNVs of terminal deletion and duplication (n = 1), CNVs <10 Mb (n = 2), or UPD (n = 1) (Table 1).

Of the 284 polyploid samples, 277 (97.5%) were triploid, and 7 (2.5%) were tetraploid. All tetraploid cases (11.7%, 7/60) were initially misdiagnosed as mosaic aneuploids below 30% involving one or more chromosomes by SNP array but confirmed as tetraploid by FISH.

Of the 504 trisomy cases associated with acrocentric chromosomes, 423 (83.9%) couples underwent chromosomal karyotyping of peripheral blood samples. Robertsonian translocations in one partner were detected in 27 couples (6.4%), 70.4% of which involved chromosomes 13 and 14 (der (13; 14) (q10; q10)).

### Large CNVs

CNVs ≥10 Mb were observed in 64 cases, including four combined with numerical abnormalities. Of the 23 cases (35.9%) with more than one CNV, 8 (34.8%) involved submicroscopic CNVs, including 3 cases with *de novo* pathogenic CNVs (No. 14 and No. 25 with haploinsufficiency regions [1p36 terminal region and 22q11.2 recurrent (DGS/VCFS) region] and No.38 with a haploinsufficiency gene [FOXC1]) (Supplementary Table S1).

### CNVs of terminal deletion and duplication

CNVs of terminal deletion and duplication were identified in 66 cases, including 5 combined with numerical chromosomal abnormalities; 61 cases (92.4%) and 5 cases (7.6%) had terminal deletion/duplication coupled with terminal duplication/deletion from different chromosomes and the same chromosome, respectively. In total, 23 cases (34.8%) had one of the terminal deletions/duplications below the traditional cytogenetic G-band analysis resolution (<10 Mb), and 1 case (No. 112; 1.5%) had both a terminal deletion and duplication below 10 Mb (Supplementary Table S2).

Overall, 14 couples declined chromosomal karyotyping of peripheral blood samples. Of the consenting couples, 29 (55.8%) had confirmed balanced chromosome rearrangements, of which 27 couples (93.1%) had reciprocal translocations and 2 (6.9%) had inversions (Supplementary Table S2).

### Submicroscopic CNVs

CNVs <10 Mb were identified in 90 cases, including 28 combined with numerical chromosomal abnormalities. In total, 97 CNVs (51 microdeletions and 46 microduplications) were detected by the SNP array, including 6 cases with multiple CNVs. Of these, 33 (34.0%) pathogenic P) CNVs and 64 (66.0%) variants of uncertain significance (VUS) were identified (Supplementary Table S3).

Furthermore, seven recurrent (n ≥ 2) CNVs were identified, including six P CNVs in 14 cases: four had a microdeletion associated with the 17p12 recurrent (HNPP/CMT1A) region, two had a microdeletion and two had a microduplication associated with the 16p11.2 recurrent region, two had a microdeletion and two had a microduplication associated with the 22q11.2 recurrent (DGS/VCFS) region, and two had a microdeletion associated with steroid sulphatase deficiency. Additionally, one VUS was identified in four cases, which was a microduplication associated with the 16p13.11 recurrent region (Supplementary Table S3).

Parental confirmation by SNP array was performed in 72.2% (65/90) of cases. The proportions of P CNVs parentally inherited or *de novo* were 47.8% (11/23) and 52.2% (12/23), respectively; for VUS, it was 74.5% (35/47) and 25.5% (12/47), respectively. Significantly more *de novo* P CNVs than *de novo* VUS CNVs were identified (*p* = 0.027) (Supplementary Table S3).

### UPD

UPD was detected in 18 cases, including four combined with numerical chromosomal abnormalities; none were from consanguineous couples. Whole-genome uniparental isodisomy (isoUPD) was observed in seven cases; all were confirmed as complete hydatidiform moles by pathological examinations. In addition, single-chromosome isoUPD (involving chromosomes 1, 4, 6, 7, 8, and 17) was identified in seven cases. Interestingly, one case of isoUPD 6 was associated with mosaic trisomy 6 (arr (6)x3 [0.46]). Finally, single-chromosome uniparental iso-heterodisomy (iso-heteroUPD; involving chromosomes 4, 11, and 17) was identified in four cases; all were maternal UPDs based on parental confirmations.

## Discussion

Miscarriage is a multifactorial condition, and advanced maternal age is the only etiological risk factor for aneuploidy (Nagaoka et al., 2012). Consistent with previous studies, more numerical chromosomal abnormalities occurred with increasing maternal age (Zhu et al., 2018). Furthermore, the frequency of normal embryonic results increased as the number of lost pregnancies increased, consistent with the findings of Ogasawara *et al.* (Ogasawara et al., 2000). Moreover, aneuploidy is more likely to cause spontaneous miscarriage than RM (Bianco et al., 2006; Campos-Galindo et al., 2015); however, we found that the proportion of RM did not differ between those with normal and abnormal results. This result likely is because most couples did not seek genetic etiology after the first spontaneous miscarriage. Several studies (Martínez et al., 2010; Qin et al., 2013) found no increased risk of chromosomal abnormalities resulting from ART (Martínez et al., 2010; Qin et al., 2013). In contrast, we observed a considerably higher rate of numerical chromosomal abnormalities after ART, but a logistic regression analysis confirmed that advanced maternal age contributed to the statistical significance.

Tetraploidy (4n) is a severe chromosomal abnormality characterized by two extra haploid sets of chromosomes, with a reported frequency of 1.4%–9.2% among first-trimester miscarriages (Gug et al., 2020). Gug *et al.* (Gug et al., 2020) reported that approximately 30% of first-trimester polyploidies detected by standard karyotype testing were tetraploids. The empirical limitation of the SNP array is that tetraploidy comprising two diploid cell lines cannot be detected (South et al., 2013; Gug et al., 2020). Notably, in our study, all seven tetraploid cases were initially misdiagnosed as mosaic aneuploids but were later confirmed to have two diploid cell lines by FISH. Additionally, we found that the incidence of tetraploidy with a 2:2 parental contribution was significantly higher than that of a 1:3 parental contribution. However, we did not perform FISH for those with negative SNP array results; thus, some tetraploids with a 2:2 parental contribution could have been missed. Therefore, we recommend FISH as a complementary technique to detect tetraploids when a negative result is detected by the SNP array, especially for mosaic aneuploids.

POC as a proband could be used to uncover the potential existence of a balanced translocation in either partner (Zhu et al., 2018). We detected 27 couples (0.8%) with Robertsonian translocations and 27 couples (0.8%) with reciprocal translocations, which is much higher than the incidence in the general population (0.1%–0.2%) (Morin et al., 2017). Couples in which one partner has a balanced translocation or inversion have an overall miscarriage risk as high as 49% due to unbalanced gametes (De Braekeleer and Dao, 1990). However, *in vitro* fertilization and preimplantation genetic testing (PGT) for structural rearrangement have significantly reduced the incidence of miscarriage for carrier couples. Moreover, parents with submicroscopic balanced translocations have a high possibility of birth with untoward outcomes rather than a first-trimester miscarriage (Morin et al., 2017). According to our results, 24 cases (36.4%) had at least one of the terminal deletions/duplications below traditional cytogenetic G-band analysis resolution. For these couples, the POC findings by CMA may help increase the positive detection rate of parental balanced translocations and the precision of the breakpoints by sub-bands. Thus, we recommend parental karyotyping after detecting CNVs of terminal deletion and duplication or aneuploids associated with acrocentric chromosomes in POCs.

Submicroscopic CNVs are considered coincidental findings in first-trimester miscarriages (Levy et al., 2014). Recently, Wang *et al.* (Wang et al., 2020) identified three recurrent submicroscopic CNVs (microdeletions in 22q11.21, 2q37.3, and 9p24.3p24.2) presumably associated with miscarriage. However, in our large-scale cohort, only two cases (0.6‰) with 22q11.21 deletion were detected, significantly lower than the previously reported prevalence (3.2%) in fetuses with congenital heart disease (Wang et al., 2018). Moreover, the small proportion (1.8%) of cases with independent submicroscopic CNVs also indicates it has a limited role in miscarriage. In addition, the frequency of P/LP CNVs in our study (0.6% [21/3,445]) was dramatically lower than that in fetuses with a normal ultrasound examination and a normal karyotype (1.7%) (Practice Bulletin No, 2016). Our findings support that large chromosomal abnormalities are more likely to be lethal, leading to miscarriage, whereas submicroscopic CNVs could result in a viable pregnancy (Wang et al., 2020). The role of P/LP CNVs in first-trimester miscarriages is controversial. However, the role of P/LP CNVs has been well-studied in patients with structural anomalies and neurodevelopmental disorders (Manning et al., 2010; Miller et al., 2010; Wapner et al., 2012). Therefore, detecting submicroscopic CNVs in POCs has crucial clinical implications because couples themselves may be carriers, with a 50% recurrent risk in all future pregnancies. Particularly, for recurrent pathogenic CNVs with incomplete penetrance and variable expressions, such as 1q21.1 distal deletions and duplications, 16p11.2 proximal and distal deletions and duplications, and 22q11.21 duplications, these CNVs can be inherited from parents with a mild or even normal phenotype (Rosenfeld et al., 2013). Thus, we recommend performing parental confirmation when P/LP CNVs are detected in POCs. PGT should be performed to improve pregnancy outcomes for these carrier couples.

In this study, we identified 18 UPD cases, which is an advantage of using the SNP array for the genetic etiology of miscarriage. The common mechanisms resulting in UPD involve trisomy rescue, monosomy rescue, and somatic mitotic crossing over (Del Gaudio et al., 2020). One limitation of the SNP array is its inability to detect hetero-UPD. However, four cases of iso-heteroUPD were detected after comparisons with the parental results. In addition, isoUPD may contain homozygosity of some lethal autosomal-recessive mutations that contribute to miscarriage, and this should be implemented in future studies. For the seven cases with whole-genome isoUPD, complete hydatidiform moles (CHMs) were simultaneously confirmed by pathological examinations. CHMs are purely androgenetic conceptions (only paternal genetic material is present) and are usually diploid (two paternal chromosome complements without a maternal chromosome complement) (Ronnett, 2018). However, most couples preferred to confirm the CHM diagnosis only by pathological examinations. Thus, we recommend SNP array and pathological examinations if CHMs are suspected.

## Conclusion

Herein, we provide an integrated laboratory algorithm for the genetic etiology of early pregnancy loss. Furthermore, we recommend an SNP array of POCs as a first-tier technique regardless of the number of miscarriages and the method of conception. However, SNP arrays have a limited range and, thus, difficulties detecting low-level mosaicism. Thus, we recommend FISH as a complementary technique to detect tetraploids if a negative SNP assay result is obtained, especially for mosaic aneuploids. Finally, we recommend parental karyotyping in cases with large CNVs, especially for those with CNVs of terminal deletion and duplication or aneuploids associated with acrocentric chromosomes. In addition, a parental SNP array should be administered if pathogenic CNVs are detected.

## Data Availability

All datasets generated for this study are included in the article/Supplementary Material. Further inquiries can be directed to the corresponding author.
