# Supplementary material for: A chromosomal microarray analysis-based laboratory algorithm for the detection of genetic etiology of early pregnancy loss
Source: Front Genet. 2023 Jun 29;14:1203891. doi: 10.3389/fgene.2023.1203891 (PMC10352453; doi:10.3389/fgene.2023.1203891)
Supplement: Supplementary file 1 [file Table2.docx]

Table S1 CNV≥10 Mb identified by CMA in 3445 POC samples

| No. | CNVs (GRCh38) | Size of CNVs (kb) | Copy number | Inherited or *de novo* |
| --- | --- | --- | --- | --- |
| 1 | arr(22)x3[0.25] 4q34.1q35.2(174901663_190036305)x3[0.31] | /  13135 | /  Gain(mosaic) | NA |
| 2 | arr(15)x3 10q22.3q26.3(78920408_133612882)x3 | /  54692 | /  Gain | NA |
| 3 | arr(10)x3[0.34] 13q31.1q34(83897588_114342257)x1[0.3] | /  30445 | /  Loss(mosaic) | NA |
| 4 | arr(21)x1[0.33] 13q22.2q34(76666272_114342258)x3[0.67] | /  37676 | /  Gain(mosaic) | NA |
| 5^[1]^ | arr (9p)x3 | / | Gain | NA |
| 6^[2]^ | arr (9p)x3[0.61] | / | Gain(mosaic) | NA |
| 7^[3]^ | arr (8p)x1  (8q)x3[0.5] | /  / | Loss Gain(mosaic) | NA |
| 8^[4]^ | arr (12p)x3[0.3]  (Y)x0[0.5] | / | Gain(mosaic) Loss(mosaic) | NA |
| 9 | arr (17p)x1 | / | Loss | NA |
| 10 | arr (18p)x1  (18q)x3 | /  / | Loss Gain | NA |
| 11 | arr (18p)x1  (18q)x3 | /  / | Loss Gain | NA |
| 12 | arr (18p)x1  (18q)x3[0.23] | /  / | Loss Gain(mosaic) | NA |
| 13 | arr (Xp)x1[1.5]  (Xq)x1 | /  / | Loss(mosaic) Loss | NA |
| 14 | 1p36.33p36.23(914087_9052538)x1 1p36.13p31.1(17731614_78465994)x3 | 8138 60734 | Loss Gain | *de novo de novo* |
| 15 | 1q43q44(236493157_248066196)x3 | 11573 | Gain | NA |
| 16 | 2q22.3q37.3(146586933_241840106)x3[0.5] | 95253 | Gain(mosaic) | NA |
| 17 | 2q24.2qq37.3(161557784__241840106)x3 | 80282 | Gain | NA |
| 18 | 2q36.3q37.3(225292724_241831406)x1[0.75] | 16539 | Loss(mosaic) | NA |
| 19 | 3p26.3p25.2(20214_12715101)x1 | 12695 | Loss | NA |
| 20 | 3p26.3p25.3(20214_11346416)x1 (11326kb)  3p25.2p24.3(12657034_20538635)x3 (7882kb) | 11326 7882 | Loss Gain | NA |
| 21 | 3p22.2p21.1(37327191_53272060)x3[0.2] | 15945 | Gain(mosaic) | NA |
| 22 | 4p16.3p15.31(68454_18982292)x3 | 18914 | Gain | NA |
| 23 | 5p15.33p14.1(113462_25035899)x1 | 24922 | Loss | NA |
| 24 | 6q25.3q27(160476860_170605209)x1 | 10128 | Loss | NA |
| 25 | 6p25.3(156975_2299006)x1 6p25.2p21.33(2645785_30572330)x3 | 2142 27927 | Loss Gain | *de novo de novo* |
| 26 | 7q31.1q36.3(108880677_159327017)x1 | 50446 | Loss | NA |
| 27 | 7q32.2q36.3(130276572_159327017)x3[0.3] | 29050 | Gain(mosaic) | NA |
| 28 | 8p23.3p21.3(277843_20154572)x1[0.24] | 19877 | Loss(mosaic) | NA |
| 29 | 8p23.3p21.3(208049_19961928)x1[0.35] | 19754 | Loss(mosaic) | NA |
| 30 | 8p23.3p23.1(208049_12633490)x1 8p23.1p12(12649549_31638864)x3 | 12425 18989 | Loss Gain | NA |
| 31 | 8p23.2p12(2531229_35645775)x1 | 33115 | Loss | NA |
| 32 | 8p23.3p11.23(208049_37178352)x1 | 36970 | Loss | NA |
| 33 | 8p23.3p23.1(208049_7141592)x1  8p23.1q24.3(12691706_145070385)x3 | 6934 132379 | Loss Gain | *de novo de novo* |
| 34 | 8p23.1p11.21(12691706_40588297)x3[0.28] 8p23.3p23.1(208049_7082691)x1 | 27897 6875 | Gain(mosaic) Loss | NA |
| 35 | 8p23.3p11.22(208049_39002790)x1 8p11.22q24.3(39008699_145070385)x3 | 38795 106062 | Loss Gain | NA |
| 36 | 9q21.11q22.2(68532723_89709725)x1 | 21177 | Loss | NA |
| 37 | 10p15.3p13(54108_16018664)x1 | 15965 | Loss | NA |
| 38 | 11p15.5q23.3(230681_116804102)x3 22q11.1q11.21(16408174_20317349)x1 | 116573 3909 | Gain Loss | *de novo de novo* |
| 39 | 11q14.3q25(88680004_135067522)x3 | 46388 | Gain | NA |
| 40 | 13q12.13q34(26066271_114342258)x3 | 88276 | Gain | NA |
| 41 | 13q14.11q14.3(42294983_54015666)x1  13q22.1q34(72912579_114342258)x1 | 11721 41430 | Loss Loss | NA |
| 42 | 13q21.1q34(56915098_114342258)x1 | 57427 | Loss | NA |
| 43 | 13q21.2q34(60746586_114342258)x1 | 53596 | Loss | NA |
| 44 | 13q21.31q34(64288783_114342258)x1  13q14.11q21.31(44324892_64281191)x4 | 50053 19956 | Loss Gain | NA |
| 45 | 13q21.33q33.3(69720350_108726708)x3 13q33.3q34(108729771_114342258)x1 | 39006 5612 | Gain Loss | NA |
| 46 | 13q31.1q34(84241103_114342258)x1 | 30101 | Loss | NA |
| 47 | 13q32.1q34(97367094_114342258)x1  13q22.1q32.1(73474046_97366782)x3 | 16975 23893 | Loss Gain | NA |
| 48 | 14q23.3(66170622_67089603)x1 16p13.3p13.11(35881_15986465)x3 | 919 15951 | Loss Gain | mat *de novo* |
| 49 | 14q31.3q32.33(85657135_106876229)x3[0.67] | 21219 | Gain(mosaic) | NA |
| 50 | 15q13.3q26.3(31965204_101888837)x3[0.47] | 69924 | Gain(mosaic) | NA |
| 51 | 15q24.1q26.3(72590173_101888837)x3 | 29299 | Gain | NA |
| 52 | 15q26.1q26.3(90075922__101751433)x1 | 11676 | Loss | NA |
| 53 | 16p13.3p12.3(35881_17285451)x3[0.68] | 17250 | Gain(mosaic) | NA |
| 54 | 16q23.1q24.3(78239382_89448459)x3 | 11209 | Gain | NA |
| 55 | 17p13.3p12(150733_11222808)x1 | 11072 | Loss | NA |
| 56 | 18p11.32p11.21(136227_15106306)x1 | 14970 | Loss | NA |
| 57 | 18p11.32p11.21(136227_13926182)x1 | 13790 | Loss | NA |
| 58 | 18q22.1q23(68601415_80255845)x1 | 11654 | Loss | NA |
| 59 | 22q12.1q13.33(25675540_50759338)x3[0.43] | 25084 | Gain(mosaic) | NA |
| 60 | Xp22.33q23(251888_110440856)x1[0.5] Xq23q28(110440856_156003433)x1 | 110189 45563 | Loss(mosaic) Loss | NA |
| 61 | Xp22.33p22.12(251888_20026186)x1 Xp22.12q22.3(20026186_107467990)x1[0.5]  Xq22.3q28(107467990_156003433)x1 | 19774 87442 48535 | Loss Loss(mosaic) Loss | NA |
| 62 | Xp22.33p11.4(251888_40100437)x1 Xp11.4q25(40100437_124969273)x1[0.5] Xq25q28(124969273_156003433)x1 | 39849 84869  31034 | Loss Loss(mosaic) Loss | NA |
| 63 | Xp22.33p21.1(251888_32928180)x1 Xp21.1q28(32929525_156003433)x3[0.41] | 32676 123074 | Loss Gain(mosaic) | NA |
| 64 | Xq26.2q28(131630070_150825398)x1[0.7] | 19195 | Loss(mosaic) | NA |

CMA: chromosomal microarray analysis; POC: product of conception; CNVs: copy number variations; NA: not available

[1], [2]: the most likely interpreted karyotype for chromosome 9 is isochromosome 9p.

[3]: the most likely interpreted karyotype is 46,X?,i(8)(q10).

[4]: the most likely interpreted karyotype for chromosome 12 is isochromosome 12p, which is associated with Pallister Killian syndrome.

Table S2 CNVs of terminal deletion and duplication identified by CMA in 3445 POC samples

| No. | CNVs (GRCh38) | Size of CNVs (kb) | Copy number | Inherited or *de novo* |
| --- | --- | --- | --- | --- |
| 65 | arr(X)x2,(Y)x1,(1-22)x3 4q35.1q35.2(183493321_189895111)x1 15q24.3q26.3(77963916_101888837)x3 | 6402 23925 | Loss Gain | paternal balanced translocation 46,XY,t(4;15)(q35;q24) |
| 66^[1]^ | arr(14)x3[0.63] 11q23.2q25(114147500_135067522)x3 22q11.1q11.21(16408174_19361391)x3 | 20920 2953 | Gain Gain | maternal balanced translocation 46,XX,t(11;22)(q23.3;q11.2) |
| 67 | 1q43q44(238741519_248930485)x1 14q24.3q32.33(74069395_106876229)x3 | 10189 32807 | Loss Gain | paternal balanced translocation 46,XY,t(1;14)(q42;q24) |
| 68 | 1q42.12q44(226591352_248930485)x1   11q23.3q25(117942522_135067522)x3 | 22339 17125 | Loss Gain | maternal balanced translocation 46,XX,t(1;11)(q42;q23) |
| 69 | 2q37.3(238938839_241840106)x1 9q33.3q34.3(123771615_141018648)x3 | 2901 17247 | Loss Gain | maternal balanced translocation 46,XX,t(2;9)(q37.2;q34) |
| 70 | 2p25.3q21.2(12771_133393337)x3  3p26.3p12.1(20214_85362012)x3 | 133381 85342 | Gain Gain | maternal balanced translocation 46,XX,t(2;3)(q21;p11) |
| 71 | 2p25.3p16.2(12771_53562119)x3  15q11.2q21.1(22582283_48253166)x1 | 53549 25671 | Gain Loss | paternal balanced translocation 46,XY,t(2;15)(p16;q21) |
| 72 | 2q11.2q37.3(97563764_241840106)x3 8q24.3(144293175_145070385)x1 | 144276 777 | Gain Loss | paternal balanced translocation 46,XY,t(2;8)(q11.2;q24.3) |
| 73 | 2q37.1q37.3(233568669_241840106)x3 4q32.1q35.2(157962909_190036305)x1 | 8271 32073 | Gain Loss | paternal balanced translocation 46,XY,t(2;4)(q37;q31) |
| 74 | 3p26.3q29(20214_193150220)x3 13q21.33q34(70150103_114342258)x3 | 193130 44192 | Gain Gain | maternal balanced translocation 46,XX,t(3;13)(q28;q21.3) |
| 75 | 3q27.2q29(185069218_196289275)x3 6q22.31q27(119012273_170605209)x1 | 11220 51593 | Gain Loss | maternal balanced translocation 46,XX,t(3;6)(q27;q22.3) |
| 76 | 5p15.33p14.1(113462_28783001)x1 8p23.3p11.22(208049_39161696)x3 | 28670 38954 | Loss Gain | paternal balanced translocation 46,XY,t(5;8)(p14;p11.2) |
| 77 | 5q34q35.3(163137660_181288095)x1 7q33q36.3(134142258_159327017)x3 | 18150 25185 | Loss Gain | maternal balanced translocation 46.XX,t(5;7)(q34;q33) |
| 78 | 5q34q35.3(163035319_181288095)x1 13q33.3q34(106708394_114342258)x3 | 18253 7634 | Loss Gain | maternal balanced translocation 46,XX,t(5;13)(q35;q33) |
| 79 | 6p25.3p21.1(156975_44286053)x3 8q24.3(144323730_145070385)x1 | 44129 747 | Gain Loss | paternal balanced translocation 46,XY,t(6;8)(p21.1;q24.3) |
| 80 | 6q27(168883080_170605209)x1 11q13.1q25(64309158_135067522)x3 | 1722 70758 | Loss Gain | maternal balanced translocation 46,XX,t(6;11)(q27;q12) |
| 81 | 7p22.3p14.1(43377_39330122)x3 13q33.1q34(101695550_114342258)x1 | 39287 12647 | Gain Loss | paternal balanced translocation 46,XY,t(7;13)(p15.1;q34) |
| 82 | 7q32.3q36.3(132059897_159327017)x3 11q23.3q25(120041960_135067522)x1 | 27267 15026 | Gain Loss | maternal balanced translocation 46,XX,t(7;11)(q32;q23.3) |
| 83 | 8p23.3p22(208049_15760720)x1 9p24.3p21.1(208455_30949768)x3 | 15553 30741 | Loss Gain | paternal balanced translocation 46,XY,t(8;9)(p22;p21) |
| 84 | 8p23.3p23.1(208049_8263311)x3 13q21.32q34(67797402_114342258)x1 | 8055 46545 | Gain Loss | maternal balanced translocation 46,XX,t(8;13)(p22;q22) |
| 85 | 8q22.2q24.3(97904977_144100759)x3 13q13.2q34(34162547_114342258)x1 | 46196 80180 | Gain Loss | maternal balanced translocation 46,XX,t(8;13)(q22;q14) |
| 86 | 8q12.2q24.3(61126695_143616750)x3 10q26.11q26.3(119458788_133612882)x1 | 82490 14154 | Gain Loss | maternal balanced translocation 46,XX,t(8;10)(q13.1;q26) |
| 87 | 8q24.21q24.3(126843691_145070385)x1 9p24.3p13.1(208455_38787483)x3 | 18227 38579 | Loss Gain | maternal balanced translocation 46,XX,t(8;9)(q24.1;p13) |
| 88 | 8q24.22q24.3(130988959_145070385)x1 13q21.2q34(60983878_114342258)x3 | 14081 53358 | Loss Gain | paternal balanced translocation 46,XY,t(8;13)(q24.2;q21.2) |
| 89 | 9p24.3p22.1(208455_18878844)x1 11p15.5p14.3(230681_25235008)x3 | 18670 25004 | Loss Gain | maternal balanced translocation 46,XX,t(9;11)(p22;p14) |
| 90 | arr(X)x2,(Y)x1,(1-22)x3 4q35.1q35.2(183493321_189895111)x1 15q24.3q26.3(77963916_101888837)x3 | 6402 23925 | Loss Gain | paternal balanced translocation 46,XY,t(4;15)(q35;q24) |
| 91 | 15q11.2q15.2(22582283_42546283)x1 22q11.1q11.21(16408174_20375099)x3 | 19964 3967 | Loss Gain | maternal balanced translocation 46,XX,t(15;22)(q15;q11.2) |
| 92 | 15q11.2q15.2(22582283_42546283)x1 22q11.1q11.21(16408174_20375099)x3 | 19964 3967 | Gain Loss | paternal balanced translocation 46,XY,t(15;22)(q15;q11.2) |
| 93 | 1p36.33p36.11(914087_24717289)x3 1q41q44(216225850_248930485)x1 | 23803 32705 | Gain Loss | paternal inversion 46,XY,inv(1)(p36.1q41) |
| 94 | arr(X)x2,(Y)x1,(1-22)x3 11q24.2q25(125687834_134916244)x1 18p11.32q11.1(136227_21187498)x1 18q11.2q23(24721827_80255845)x3 20q11.1q13.33(30275344_64282292)x3 | 9228 21051 55534 34007 | Loss Loss Gain Gain | *de novo* |
| 95 | arr(18)x3 6q25.2q27(154296376_170605209)x1 9p24.3p21.1(208455_31244988)x3 | 16309 31037 | Loss Gain | NA |
| 96 | arr(21)x3 13q22.2q34(75428277_114342258)x1 14q24.3q32.33(76583217_106876229)x3 | 38914 30293 | Loss Gain | *de novo* |
| 97 | 1p36.33p36.12(914087_20336445)x1 5p15.33p13.2(113462_37065291)x3 | 19422 36952 | Loss Gain | NA |
| 98 | 1p36.33p36.22(914087_10809661)x1 1q42.13q44(228081278-248773355)x3 | 9896 20692 | Loss Gain | NA |
| 99 | 1q41q44(219274411_248930485)x3 6q25.3q27(158027915_170605209)x3 | 29656 12577 | Gain Gain | *de novo* |
| 100 | 1q42.3q44(235445946_248930485)x3 9p24.3p24.2(208455_4207357)x1 | 13485 3999 | Gain Loss | *de novo* |
| 101 | 2p25.3p23.1(12771_30367908)x3 8p23.3p21.2(208049_27380598)x1 | 30355 27173 | Gain Loss | *de novo* |
| 102 | 2p25.3q31.1(12771_169797530)x3 13q32.3q34(99234094_114248706)x3 | 169785 15015 | Gain Gain | *de novo* |
| 103 | 3q26.31q29(175499065_198124573)x3 13q31.1q34(84574457_114342258)x1 | 22626 29768 | Gain Loss | NA |
| 104 | 3q26.32q29(176712786_198124573)x3 Xq22.3q28(108818043_155967226)x1 | 21412 47149 | Gain Loss | *de novo* |
| 105 | 3q26.31q29(171960151_198124573)x3 4q32.1q35.2(159113183_190036305)x1 | 26164 30923 | Gain Loss | *de novo* |
| 106 | 3q27.2q29(185653884_196043138)x3 9q34.3(137334671_138124196)x1 | 10389 790 | Gain Loss | *de novo* |
| 107 | 4p16.3p15.32(68454_16661058)x1 4q32.1q35.2(159576084_190036305)x1 | 16593 30460 | Loss Loss | *de novo* |
| 108 | 4p16.3p15.1(68454_32790342)x1 4q34.3q35.2(182156904_190036305)x3 | 32722 7879 | Loss Gain | *de novo* |
| 109 | 4p16.3(68454_4442972)x3 9p24.3p22.2(208455_17078747)x1 | 4375 16870 | Gain Loss | *de novo* |
| 110 | 4p16.3p15.31(68454_19507848)x1 7q21.12q36.3(87089284_159327017)x3 | 19439 72238 | Loss Gain | *de novo* |
| 111 | 4p16.3p15.1(68454_29313916)x1 10q25.2q26.3(112658923_133612882)x3 | 29245 20954 | Loss Gain | NA |
| 112 | 4q34.3q35.2(182095610_190036305)x1 15q26.1q26.3(92754336_101888837)x3 | 7941 9135 | Loss Gain | NA |
| 113 | 4q34.1q35.2(174323704_190036305)x1 11q13.2q25(67656439_134937416)x3 | 15713 67281 | Loss Gain | NA |
| 114 | 5p15.33p15.31(113462_6672234)x1 5q23.2q35.3(123758538_181288095)x3 | 6559 57530 | Loss Gain | *de novo* |
| 115 | 5q14.3q35.3(91420861_181288095)x3 18q21.2q23(55308208_80255845)x1 | 89867 24948 | Gain Loss | NA |
| 116 | 6q25.1q27(150977873_170605209)x3 8p23.3p12(208049_31770894)x1 | 19627 31563 | Gain Loss | NA |
| 117 | 6q25.1q27(151018995_170605209)x3 8q22.2q24.3(99817171_143741789)x3[0.32] 18p11.32p11.22(136227_8936311)x1 | 19586 43925 8800 | Gain Gain(mosaic) Loss | *de novo* |
| 118 | 7q11.21q36.3(64027951_159119707)x3 10q26.13q26.3(122475780_133612882)x1 | 95092 11137 | Gain Loss | *de novo* |
| 119 | 7q21.11q36.3(85995248_159327017)x3 8p23.3p12(208049_36698935)x1 | 73332 36491 | Gain Loss | *de novo* |
| 120 | 8p23.3p12(208049_31274218)x3 18p11.32p11.21(136227_15181209)x1 | 31066 15045 | Gain Loss | *de novo* |
| 121 | 8q22.1q24.3(96293129_145070385)x3 12p13.33p11.1(94423-31849235)x1 | 48777 31755 | Gain Loss | NA |
| 122 | 8q24.12q24.3(118317217_145070385)x1 17q24.2q25.3(66684891_83083947)x3 | 26753 16399 | Loss Gain | *de novo* |
| 123 | 9p13.3q34.3(34039640_138124196)x3 10p15.3p12.1(107835_26822349)x3 | 104085 26715 | Gain Gain | *de novo* |
| 124 | 10q23.33q26.3(94169620_133612882)x3 21q11.2q22.3(13644166_46673449)x1 | 39443 33029 | Gain Loss | NA |
| 125 | 11q14.3q25(90043665_134937416)x3 18q22.1q23(64306713_80255845)x1 | 44894 15949 | Gain Loss | NA |
| 126 | 11q22.1q24.3(99900774_129642604)x3 11q24.3q25(129649820_135067522)x1 20p13p12.1(81022_13161162)x3 | 29742 5418 13080 | Gain Loss Gain | *de novo* |
| 127 | 12q24.33(132494880_133200976)x1 22q12.2q13.33(30523129_50759338)x3 | 706 20236 | Loss Gain | *de novo* |
| 128 | 16q21q24.3(66423858_90088654)x3 22q13.2q13.33(41417910_50759338)x1 | 23665 9341 | Gain Loss | NA |
| 129 | 18p11.32p11.21(136227_14598139)x1 21q11.2q22.3(13644166_46673449)x3 | 14462 33029 | Loss Gain | NA |
| 130 | 18q11.2q23(25160235_80255845)x3[0.77] 18q22.3q23(73029299_80255845)x1 Xq27.3q28(143944323_156003433)x3 | 55096 7227 12059 | Gain(mosaic) Loss Gain | *de novo* |

CMA: chromosomal microarray analysis; POC: product of conception; CNVs: copy number variations; NA: not available

[1]: the most likely interpreted karyotype is a supernumerary derivative chromosome 22 [der(22)] involved a duplication of 22q10-22q11 and duplication of 11q23-qter, which is associated with Emanuel syndrome.

Table S3 CNVs<10 Mb detected by CMA among the 3445 POC samples

| No. | CNVs (GRCh38) | Size of CNVs (kb) | Copy number | HITS region | HITS gene | ACMG Classification | Inherited or *de novo* |
| --- | --- | --- | --- | --- | --- | --- | --- |
| 131 | arr(16)x3 15q11.2(22582283_23380638)x1 | 798 | Loss | /  15q11.2 recurrent region (BP1-BP2) (includes NIPA1) | /  / | /  P | *de novo*  pat |
| 132 | arr(16)x3 15q25.2(82330010_84165371)x1 | 1835 | Loss | /  15q25.2 recurrent region (proximal LCR B-LCR C) | /  / | /  P | NA |
| 133 | arr(16)x3 15q26.1q26.2(93292938_94008167)x1 | 715 | Loss | /  / | /  CHD2 | /  P | NA |
| 134 | arr(22)x3 16p11.2(29417211_30165187)x1 | 748 | Loss | /  16p11.2 recurrent region (proximal, BP4-BP5) (includes TBX6) | /  / | /  P | NA |
| 135 | arr(15)x3 16p11.2(29580006_30291027)x3 | 711 | Gain | /  16p11.2 recurrent region (proximal, BP4-BP5) (includes TBX6) | /  / | /  P | *de novo*  *de novo* |
| 136 | arr(22)x3 17p12(14170219_15518221)x3 | 1348 | Gain | /  17p12 recurrent (HNPPCMT1A) region (includes PMP22) | /  / | /  P | *de novo*  mat |
| 137 | arr(X,1-22)x3 22q11.21(18947951_19697386)x4 | 749 | Gain | /  22q11.2 recurrent (DGSVCFS) region (proximal, A-D) (includes TBX1) | /  / | /  P | NA |
| 138^[1]^ | arr(22)x3[0.23] 22q11.1q11.21(16408174_19598958)x3 | 3191 | Gain | /  22q11.21 recurrent (Cat eye syndrome) region (includes CECR2) | /  / | /  P | NA |
| 139 | arr(X,1-22)x3 Xp22.33(1714020_4362056)x2 | 2648 | Loss | /  / | /  ARSL | /  P | *de novo*  *de novo* |
| 140 | arr(16)x3 Xp22.31(6549860_7893624 )x1 | 1344 | Loss | /  / | /  STS | /  P | *de novo*  mat |
| 141 | arr(8)x3 Xp21.1(31613916_31905655)x1 | 292 | Loss | /  / | /  DMD | /  P | *de novo*  mat |
| 142 | 1p36.33p36.22(914087_9271975)x1[0.30] | 8358 | Loss  (mosaic) | 1p36 terminal region (includes GABRD) | CAMTA1 | P | NA |
| 143 | 1q32.2q41(209406530_214644459)x1 | 5238 | Loss | / | IRF6 | P | *de novo* |
| 144 | 2q37.2q37.3(234942103_241840106)x1 | 6898 | Loss | 2q37.3 terminal region (includes HDAC4) | / | P | NA |
| 145 | 4p16.3(68454_1885333)x1 | 1817 | Loss | 4p16.3 terminal (Wolf-Hirschhorn syndrome) region | / | P | *de novo* |
| 146 | 6p25.3p25.2(156975_4062553)x1 | 3906 | Loss | / | FOXC1 | P | *de novo* |
| 147 | 7q11.23(73250431_74761235)x3 | 1511 | Gain | 7q11.23 recurrent (Williams-Beuren syndrome) region (includes ELN) | / | P | *de novo* |
| 148 | 8p23.1(7141698_12633490)x1 | 5492 | Loss | / | GATA4 | P | *de novo* |
| 149 | 15q26.2q26.3(94701920_101888837)x1 | 7187 | Loss | / | IGF1R | P | *de novo* |
| 150 | 15q13.2q13.3(30094195_32623522)x1  22q11.21(18929330_21110475)x1 | 2529  2181 | Loss  Loss | 15q13.3 recurrent region (BP4-BP5) (includes CHRNA7) 15q13.3 recurrent region (D-CHRNA7 to BP5) (includes CHRNA7, OTUD7A)  22q11.2 recurrent (DGSVCFS) region (proximal, A-D) (includes TBX1) | /  / | P  P | *de novo*  *de novo* |
| 151 | 16p11.2(29580006_30319560)x3 | 740 | Gain | 16p11.2 recurrent region (proximal, BP4-BP5) (includes TBX6) | / | P | pat |
| 152 | 16p11.2(29555975_30165187)x1 | 609 | Loss | 16p11.2 recurrent region (proximal, BP4-BP5) (includes TBX6) | / | P | mat |
| 153 | 2q13q14.1(109740565_112354279)x1 17p11.2(16853302_20530189)x1 | 2614 3677 | Loss Loss | 17p11.2 recurrent (SMSPLS) region (includes RAI1) | FLCN/  RAI1 | VUS P | pat *de novo* |
| 154 | 17q22q24.2(58932622_68969984)x1 | 10037 | Loss | 17q23.1q23.2 recurrent region (includes TBX2, TBX4) | TBX4/  BRIP1/  AXIN2 | P | NA |
| 155 | 17p12(14182336_15587795)x1 | 1405 | Loss | 17p12 recurrent (HNPPCMT1A) region (includes PMP22) | PMP22 | P | pat |
| 156 | 17p12(14196248_15609739)x1 | 1413 | Loss | 17p12 recurrent (HNPPCMT1A) region (includes PMP22) | PMP22 | P | mat |
| 157 | 17p12(14196248_15579519)x1 | 1383 | Loss | 17p12 recurrent (HNPPCMT1A) region (includes PMP22) | PMP22 | P | mat |
| 158 | 17p12(14196248_15579519)x1 | 1383 | Loss | 17p12 recurrent (HNPPCMT1A) region (includes PMP22) | PMP22 | P | mat |
| 159 | 22q11.21(18989177_21110475)x3 | 2121 | Gain | 22q11.2 recurrent (DGSVCFS) region (proximal, A-D) (includes TBX1) | / | P | NA |
| 160 | 22q11.21(18153983_21110475)x1 | 2956 | Loss | 22q11.2 recurrent (DGSVCFS) region (proximal, A-D) (includes TBX1) | / | P | *de novo* |
| 161 | 22q13.2q13.33(41784016_50759338)x1 | 8975 | Loss | / | SHANK3/  TCF20 | P | NA |
| 162 | Xp22.31(7342952_7634488)x1 | 292 | Loss | / | STS | P | mat |
| 163 | arr(22)x3 1q21.2(148263622_148731429)x1 | 468 | Loss | /  / | /  / | /  VUS | *de novo*  *de novo* |
| 164 | arr(7)x3,(14)x3 1q42.3q43(235570808_237038763)x3 | 1468 | Gain | /  / | /  / | /  VUS | *de novo*  pat |
| 165 | arr(16)x3 1q44(247763420_248725011)x1 | 962 | Loss | /  / | /  / | /  VUS | *de novo*  pat |
| 166 | arr(X)x1 2p14(66784373_68139088)x3 | 1355 | Gain | /  / | /  / | /  VUS | *de novo*  mat |
| 167 | arr(5)x3 2q21.1q21.2(131290840_132493772)x1 | 1203 | Loss | /  / | /  / | /  VUS | NA |
| 168 | arr(X)x1 3q12.3q13.12(102932521_107663555)x3 | 4731 | Gain | /  / | /  / | /  VUS | *de novo*  *de novo* |
| 169 | arr(16)x3 3q26.31(173454327_174271669)x1 | 817 | Loss | /  / | /  / | /  VUS | *de novo*  pat |
| 170 | arr(5)x3 4p14p13(40636428_42719308)x3 | 2083 | Gain | /  / | /  / | /  VUS | NA |
| 171 | arr(4)x3[0.31] 4q12(51823360_56062958)x3~4 | 4240 | Gain | /  / | /  / | /  VUS | *de novo*  *de novo* |
| 172 | arr(6)x3,(8)x3 7p21.3(8770028_11122788)x3 | 2353 | Gain | /  / | /  / | /  VUS | *de novo*  NA |
| 173 | arr(16)x3,(22)x3 8q22.3q23.1(103505745_105363808)x3 | /  1858 | /  Gain | /  / | /  / | /  VUS | *de novo*  pat |
| 174 | arr(14)x3 9p24.3(629289_1727478)x3 | 1098 | Gain | /  / | /  / | /  VUS | NA |
| 175 | arr(X)x1 10p15.3p15.2(1162411_3092489)x3 | 1930 | Gain | /  / | /  / | /  VUS | NA |
| 176 | arr(X)x1,(21)x3 14q21.2(45167110_46294061)x3 | 1127 | Gain | /  / | /  / | /  VUS | *de novo*  mat |
| 177 | arr(9)x3 15q23(70290002_71576412)x3 | 1286 | Gain | /  / | /  / | /  VUS | *de novo*  mat |
| 178 | arr(10)x3 18q22.3(72762039_74502801)x3 | 1741 | Gain | /  / | /  / | /  VUS | *de novo*  mat |
| 179 | arr(16)x3 22q11.23(23222342_25453958)x3 | 2232 | Gain | /  / | /  / | /  VUS | NA |
| 180 | 16p13.11(15416656_16184275)x3 | 768 | Gain | 16p13.11 recurrent region (BP2-BP3) (includes MYH11) (HI score: 2) | / | VUS | pat |
| 181 | 16p13.11(14835214_16169504)x3 | 1334 | Gain | 16p13.11 recurrent region (BP2-BP3) (includes MYH11)(HI score: 2) | / | VUS | NA |
| 182 | 12p13.33(64621_1871781)x1 16p13.11(14799119_16444739)x3 | 1807 1646 | Loss Gain | /  16p13.11 recurrent region (BP2-BP3) (includes MYH11)(HI score: 2) | /  / | VUS VUS | mat pat |
| 183 | 16p13.11(14964966_16215189)x3 | 1250 | Gain | 16p13.11 recurrent region (BP2-BP3) (includes MYH11)(HI score: 2) | / | VUS | pat |
| 184 | 1p21.3(94546329_95583745)x3 | 1037 | Gain | / | / | VUS | *de novo* |
| 185 | 2q13(109342662_110222718)x3 | 880 | Gain | / | / | VUS | NA |
| 186 | 2q32.3(193509938_194518231)x3 | 1008 | Gain | / | / | VUS | pat |
| 187 | 2q34(211743818_212952909)x3 | 1209 | Gain | / | / | VUS | mat |
| 188 | 2q37.2(234717583_236103287)x3 | 1386 | Gain | / | / | VUS | *de novo* |
| 189 | 3p26.3(1393762_2584178)x3 | 1190 | Gain | / | / | VUS | mat |
| 190 | 4q23(99336998_99658708)x3 | 322 | Gain | / | / | VUS | pat |
| 191 | 4q24(105556663_106696615)x1 | 1140 | Loss | / | / | VUS | *de novo* |
| 192 | 4q33q34.1(170174988_171113227)x1 | 938 | Loss | / | / | VUS | NA |
| 193 | 4q35.2(188545292_190036305)x1 | 1491 | Loss | / | / | VUS | pat |
| 194 | 5p15.33p15.31(113462_7114154)x1 | 7001 | Loss | / | / | VUS | NA |
| 195 | 5p15.2(12448334_13415573)x1 11q24.2q25(125739758_131652322)x3 11q25(131658607_135067522)x1 | 967 5913 3409 | Loss Gain Loss | /  /  / | /  /  / | VUS VUS VUS | NA |
| 196 | 5p14.3(18797480_19432032)x1 | 635 | Loss | / | / | VUS | pat |
| 197 | 5p15.2(12448334_13249376)x1 | 801 | Loss | / | / | VUS | pat |
| 198 | 6p22.3(18922028_21222478)x1 | 2300 | Loss | / | / | VUS | pat |
| 199 | 6q13(69472241_70482359)x3 | 1010 | Gain | / | / | VUS | mat |
| 200 | 6q27(167969824_170504006)x1 | 2534 | Loss | / | / | VUS | *de novo* |
| 201 | 8p23.3p23.2(2275006_6278991)x3 | 4004 | Gain | / | / | VUS | mat |
| 202 | 8p23.1p22(12691706_13873637)x3 | 1182 | Gain | / | / | VUS | pat |
| 203 | 8p23.1p22(12691706_13807983)x3 | 1116 | Gain | / | / | VUS | pat |
| 204 | 8p23.2(3032370_5467572)x3 | 2435 | Gain | / | / | VUS | mat |
| 205 | 8q22.3(101321688_102229302)x1 | 908 | Loss | / | / | VUS | mat |
| 206 | 9p21.1(28649118_32306047)x1 | 3657 | Loss | / | / | VUS | mat |
| 207 | 10q26.2q26.3(126895324_130644629)x3 10q26.3(132825790_133612882)x1 | 3749 787 | Gain Loss | / | / | VUS VUS | *de novo de novo* |
| 208 | 11q14.3q21(91437162_93086351)x3 | 1649 | Gain | / | / | VUS | mat |
| 209 | 11q22.1q22.3(98873147_103851913)x3 | 4979 | Gain | / | / | VUS | NA |
| 210 | 12p13.33p13.32(64621_3680783)x3 | 3616 | Gain | / | / | VUS | NA |
| 211 | 12q14.1(59474072_60202070)x1 | 728 | Loss | / | / | VUS | pat |
| 212 | 13q33.3(109039640_109528658)x1 | 489 | Loss | / | / | VUS | mat |
| 213 | 13q34(109817054_110930649)x3 | 1114 | Gain | / | / | VUS | pat |
| 214 | 16q23.1(77889109_79019713)x3 | 1131 | Gain | / | / | VUS | NA |
| 215 | 17p13.3(150733_813206)x1 | 662 | Loss | / | / | VUS | mat |
| 216 | 18q22.3q23(73958182_80255845)x1 | 6298 | Loss | / | / | VUS | NA |
| 217 | 19p13.3(3501743_5750514)x1 | 2249 | Loss | / | / | VUS | *de novo* |
| 218 | 21q22.3(43572694_44821768)x3 21q22.3(44827502_46673449)x1 | 1249 1846 | Gain Loss | / | / | VUS VUS | *de novo de novo* |
| 219 | Xq23(112643833_114447439)x3 | 1804 | Gain | / | / | VUS | mat |
| 220 | Yq11.223q11.23(23617871_25959332)x0 | 2341 | Loss | / | / | VUS | pat |

CMA: chromosomal microarray analysis; POC: products of conception; CNVs: copy number variations; HI: haploinsufficiency; TS: triplosensitivity; P: pathogenic;

LP: likely pathogenic; VUS: variations of uncertain significance; NA: not available

[1]: the most likely interpreted karyotype is a small supernumerary marker chromosome involved tetrasomy/triplication (four copies) of the proximal region of chromosome 22q, which is associated with Cat eye syndrome.
